# Event Detection using Twitter: A Spatio-Temporal Approach

**DOI:** 10.1371/journal.pone.0097807

**Published:** 2014-06-03

**Authors:** Tao Cheng, Thomas Wicks

**Affiliations:** SpaceTimeLab, Department of Civil, Environmental and Geomatic Engineering, University College London, London, United Kingdom; Kenya Medical Research Institute (KEMRI), Kenya

## Abstract

**Background:**

Every day, around 400 million tweets are sent worldwide, which has become a rich source for detecting, monitoring and analysing news stories and special (disaster) events. Existing research within this field follows key words attributed to an event, monitoring temporal changes in word usage. However, this method requires prior knowledge of the event in order to know which words to follow, and does not guarantee that the words chosen will be the most appropriate to monitor.

**Methods:**

This paper suggests an alternative methodology for event detection using space-time scan statistics (STSS). This technique looks for clusters within the dataset across both space and time, regardless of tweet content. It is expected that clusters of tweets will emerge during spatio-temporally relevant events, as people will tweet more than expected in order to describe the event and spread information. The special event used as a case study is the 2013 London helicopter crash.

**Results and Conclusion:**

A spatio-temporally significant cluster is found relating to the London helicopter crash. Although the cluster only remains significant for a relatively short time, it is rich in information, such as important key words and photographs. The method also detects other special events such as football matches, as well as train and flight delays from Twitter data. These findings demonstrate that STSS is an effective approach to analysing Twitter data for event detection.

## Introduction

Since its launch in 2006, Twitter has become one of the internet’s most popular microblogging sites [1]. Its fundamental premise relies on users sending messages, known as ‘tweets’, into a hyper-real digital space, known as the Twittersphere [2], [3]. This concept has proven to be highly successful, with over 400 million tweets sent worldwide each day [4]. Additionally, the advent of GPS-enabled mobile platforms has augmented tweets, which were previously only temporally annotated, with spatial information. Accordingly, Twitter is emerging as a key resource of free and open volunteered geographic information (VGI; [5]). While much of this data is noise, containing non-descript communication and chatter, some tweets contain attempts at citizen journalism [6], whereby users describe and provide information about the world around them.

This data is beginning to be used as a basis for detecting, monitoring and analysing the characteristics of both natural and man-made disasters. Currently, the literature relevant to Twitter and disaster events uses a volumetric method of analysis. Accordingly, present methodologies choose a selection of words and hashtags to follow during an event, with tweets containing the selected words being deemed relevant to the event. For example, recent papers monitor tweets containing the word “earthquake” and other related words [7], [8]. They assume that a spike in earthquake-related tweets correlates with an earthquake event. In doing this, 75% of earthquakes are detected by Twitter within two minutes and, as such, outpace traditional geological survey detections [8].

Similarly, this methodology of selecting tweets has been applied within a variety of other disaster events, using the same technique to select tweets containing key words relevant to the 2009 Oklahoma fires, such as ‘Oklahoma’, ‘grassfire’ and ‘OKfire’, in order to study retweeting conventions during mass emergencies [9]. Others utilise tweets containing the hashtag ‘#qldfloods’ to analyse the 2011 flooding event in Queensland, Australia [10]. Similar studies analyse two separate hurricane events by utilising tweets containing the word ‘hurricane’ as well as the respective hurricane names [11].

However, the methodology described generates several issues which severely hamper the results of these studies. Perhaps most critically, following tweets containing only a select set of words means that many other tweets relevant to the disaster event are missed out entirely from the study. Additionally, the choice of words to follow is subjective based on the authors’ perception of the event. Furthermore, it requires some prior knowledge of the event in question in order to know which words to track, preventing instantaneous data collection from the very moment of impact. Moreover, this method assumes that, during disasters, Twitter users are independent and identically distributed [7]. This assumption means that if a user tweets about an earthquake, their followers are no more likely than normal to also tweet about an earthquake. In reality, this assumption is unlikely to be true, which may lead to spurious results.

Here the method of space-time scan statistics (STSS) is introduced as a differing analytical technique which overcomes some of the challenges faced by existing methodological frameworks for event detection from Twitter data. This technique looks for clusters within the dataset across both space and time, regardless of tweet content. It is expected that clusters of tweets will emerge during spatio-temporally relevant events, as people will tweet more than expected in order to describe the event and spread information.

The STSS technique is applied to a case study of the 2013 London helicopter crash. Its performance in detecting space-time events is also evaluated for finding other events (including football games and travel delays) at on an hourly or daily basis. It is shown in the case study that, by using STSS, tweets can be more appropriately selected and utilised as a live source of information on (disaster) events compared to current methods. This is found to be true, with spatio-temporally significant clusters found to exist relating to the 2013 London helicopter crash.

## Methods

### 2.1 Space Time Scan Statistics

In order to achieve a more appropriate method for detecting disaster events, a technique is required to identify the space-time locations of tweet clusters. One method that achieves this is a space-time scan statistic, implemented via the SatScan™ 9.0 software. STSS were first applied to epidemiological settings [12], but have since been used in a variety of contexts, such as crime [13], [14], forest fires [15] and construction [16].

In principle, STSS views data points, known as incidences, within a space-time cube. It then goes on to move a cylindrical window, of varying radius (space) and height (time), across the study area [17]. This process is repeated until all possible space-time locations have been visited. Each window is then viewed as a potential cluster; with the number of incidences within each being compared to the number of expected incidences for that window. This identifies cylinders which possess a greater than expected number of incidences. Each cluster’s significance is then tested, giving each a p-value, describing the likelihood that it occurred by chance. For this study, p-values were generated using Gumbel approximation due to the fact that it requires less processing time than other methods and can produce small p-values with no lower limit beyond *p*>0 [18]. It is thus suited to large datasets such as those provided by Twitter.

It is important to note that STSS can be used either retrospectively or prospectively. Retrospective analysis looks for clusters across all possible time periods [12]. As such a cluster may have occurred at the very beginning of the data, at the very end or any period and length in-between. Accordingly, retrospective analysis discovers historic clusters. On the other hand, prospective analysis only looks for ‘alive clusters’ [12], meaning a cluster can last any period of time, but must be ongoing within the most recent time period. As this paper aims to look at the suitability of STSS as a methodological tool for Twitter analysis, retrospective analysis was used, in order to test whether the method can detect historic disaster events.

**Figure 1:**
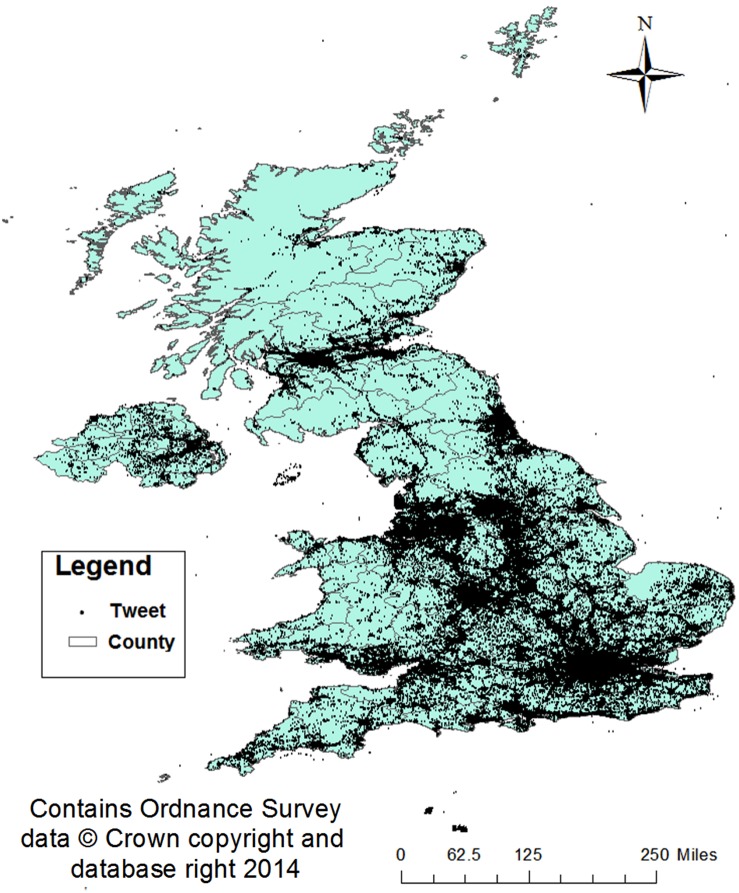
The locations of tweets collected between 7th January and 18th January 2013.

**Figure 2:**
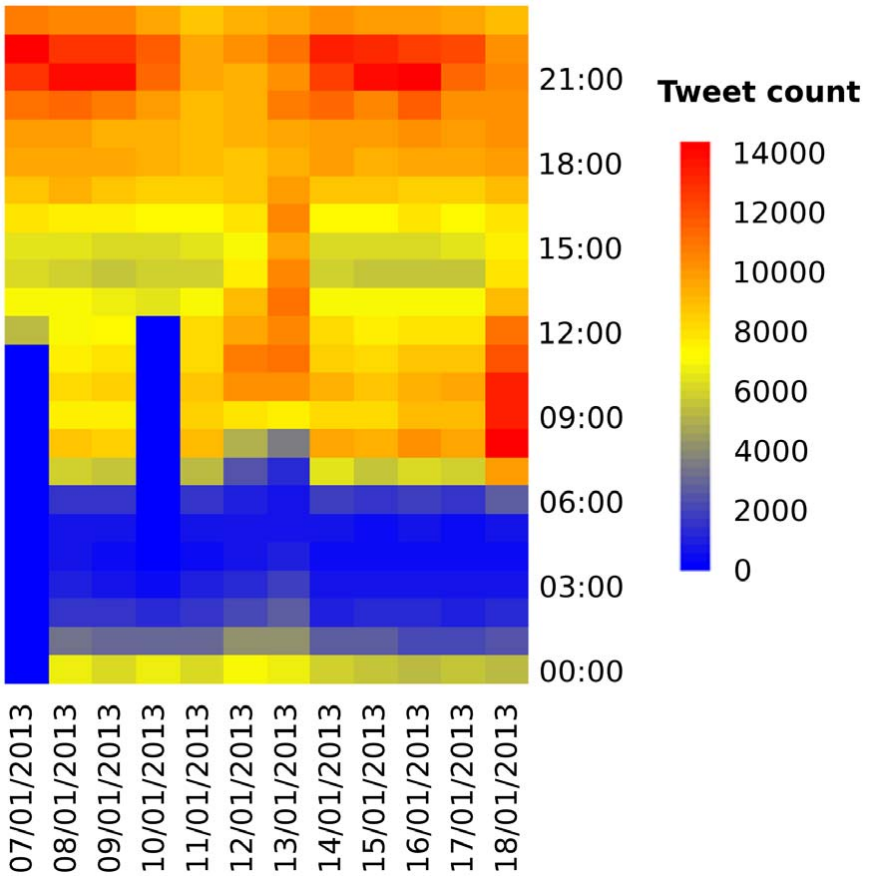
The temporal distribution of tweets collected between 7th January and 18th January 2013.

Furthermore, a variety of STSS models are available for analysis, such as the Bernoulli model, the Poisson model and the permutation model. Each model’s use varies with the context within which the data is being used. For this analysis, a space-time permutation model (STPM) was used due to its flexibility when compared to other models. The model simply requires data to contain spatial and temporal attributes and requires no further information, thus matching the attributes collected by each tweet. Additionally, the STPM automatically allows for purely spatial and purely temporal variations in a dataset. This is a critical feature for Twitter data analysis given the large temporal and spatial variations on the data, as illustrated by figures 1 and 2. A detailed methodology of the STPM goes beyond the scope of this paper but is provided in [19].

### 2.2 Latent Dirichlet Allocation

Having used a STPM for cluster detection, a method is required to evaluate whether clusters relate to space-time events. In order to do this, tweets must be classified into groups of topics that describe their content. This could be undertaken manually, by reading tweets and classifying them into topics. However, this would be subjective and take a considerable amount of time.

Instead, it is possible to use unsupervised learning algorithms, such as Latent Dirichlet Allocation (LDA), to classify tweets into topics. These topics can then be analysed to see what the general theme for each cluster is. LDA was first introduced for general text classification [20] and has subsequently been applied to classifying Twitter data [21]–[24]. LDA builds on the “bag of words” approach [25], where each sentence is seen to contain a set of words. The frequency of these words is then extracted to create a probability distribution of key terms that are likely to be found in each topic. This distribution goes on to provide a probabilistic list of key terms that define each topic [24]. Accordingly, within this study, clusters are classified by the terms contained within each cluster’s topics, with those found to represent space-time events being used for further analysis. Four topics will be searched for within each significant space-time cluster. This number of topics has been chosen to align with other research, such as [24], which also uses four topics during tweet classification. LDA was conducted using R version 3.0.1 and the “RTextTools” [26] and “topicmodels” [27] packages.

### 2.3 Data Collection

Data are acquired through the use of the Twitter streaming application programming interface (API) [28], which offers a live feed of tweets to be downloaded free of charge [29]. Moreover, it allows filtering of the live stream using a variety of parameters [30]. This project only downloads tweets that originate from geo-enabled users within a bounding box set of coordinates containing the UK. Each tweet contained attributes such as the latitude and longitude of where the tweet was sent, the time it was sent, the tweet content and the tweet originator’s username.

The use of Twitter’s streaming API suffers from some limitations. The API only allows a maximum of 1% of the available Twitter stream to be freely downloaded [31]. As such, the vast majority of tweets are actually never collected with this method. Moreover, only 1%, of users geo-tag their tweets [32]. This is because this service is ‘opt-in’ rather than set as a default. Yet, as roughly 1% of tweets are geo-enabled, the vast majority are downloaded by the 1% allowance of the API when filtering by location (i.e. the 1% downloaded are the 1% of geo-enabled tweets) [31]. As such, a fairly comprehensive sample of geo-tagged tweets is collected.

Additionally, it is important to remember that Twitter is an inherently biased data source due to the demographics of its users. For example, when looking at the age of users, 26% are under 22 years old, while 60% are under 35 [33]. While it is important to bear this in mind, for the purposes of this research there will be no impact on the types of events detected if we assume the group of people share a consistent behaviour of using Twitter to communicate with their friends or share the news. Furthermore, it is assumed that disaster events make significant emotional impact on all people, regardless of their demographics. Accordingly, there should be minimal bias within the dataset when it comes to Twitter reporting significant events.

### 2.4 The Case

At 7∶59 am on the 16^th^ January 2013, a helicopter crashed into a crane attached to St. George’s Warf Tower, Vauxhall [34]. Freezing fog had drastically reduced visibility levels, causing the pilot to divert from the planned route and lower his altitude. Upon hitting the crane, debris was scattered over the surrounding area. A section of crane fell onto Nine Elms Lane, while the helicopter crashed onto Wandsworth Road [34]. During the incident, the pilot was killed, along with a pedestrian [34]. A further 12 people sustained injuries [35].

In order to study this event, data were collected between the 7^th^ January 2013 and 18^th^ January 2013. This generated 1,852,700 unique tweets from the UK, with 183,731 originating from Greater London. This date range was chosen in order to collect tweets from the week before and two days after the crash. A longer date range after the crash was not possible due to the Twitter API server connection failing between 19^th^ January and 21^st^ January 2013 inclusive.

## Results

### 3.1 Exploratory Data Analysis

Figure 1 maps the spatial extent of tweets collected from within the United Kingdom. As can be seen, tweets are dispersed throughout the region. However, the majority of tweets are generated from within major urban conurbations such as London, Birmingham and Manchester. Additionally, major arterial road and train lines can be seen as areas which generate high tweet volumes.

Figure 2 explores the temporal dimensions of the tweets collected. As can be seen, there are clear daily fluctuations in tweet volumes generated within the UK. It should be noted that during the mornings of the 7th January and 10th of January, the server connection to the Twitter API failed and accordingly no tweets were collected during this time. Yet, clear patterns can be seen from this graph. First, the early morning period (between 1 AM and 6 AM) consistently sees very few tweets generated. Secondly, the morning period (7 AM to 12 PM) sees a relatively high volume of tweets being sent. Next, the afternoon period (2 PM to 4 PM) sees a fall in the number of tweets generated within the UK. Lastly, the evening period (5 PM to 11 PM) sees a large increase in Twitter activity, particularly around 9 PM and 10 PM.

However, two anomalies differ from these patterns. The days of 12th January and 13th January exhibit a substantially different tweet pattern to the rest of the days sampled. It is likely that this is due to these days being weekends. These days see an elongated early morning peak with few tweets sent, with a large volume during the afternoon. The number of evening tweets is then considerably lower than other days. Moreover, the morning of the 18th January saw a large increase in the number of tweets sent. It is likely that this is down to a large overnight snowstorm which hit much of the UK causing unexpectedly large tweet volumes.

### 3.2 Space-time Scan Statistic Analysis

Due to the large volume of Twitter data, it is necessary to aggregate tweets into small periods, such as days and hours. Using a longer time period, such as days means a larger amount of data can be used for analysis. Using smaller time periods, such as hours means smaller datasets must be used to meet memory requirements. In this paper we study two different period lengths to assess which is best applied to Twitter. The search is looking for very localized and time specific events, so small maximum temporal and spatial cluster length would seem appropriate. However, it was not possible to determine *ab initio* how spatially temporally localised the event would be. Selecting lower values may artificially reduce the apparent spatial extent of the clusters. Therefore, the maximum temporal size was 50% of the period and the maximum spatial size was 50% of the length of the region considered (these are the default values of the SatScan software).

Table 1 outlines the different periods used. When analysing the data with these two varying aggregations, differing numbers of clusters are found. Table 2 summarises the results generated by SatScan and reports the number of clusters found. As can be seen, large numbers of clusters are found for both time frames. It is expected that the majority of these clusters contain noise, and are not attributable to any event in particular. However, this theory cannot be proven until clusters are classified. Interestingly, using hourly aggregation generated the highest number of significant clusters, but a substantially lower number of overall clusters when compared to daily aggregations. This suggests that while fewer clusters exist using this temporal aggregation, more of those that do exist are significant than when using daily aggregations.

**Table 1:** The two temporal aggregations used during space-time scan statistics.

Time Frame	Temporal Aggregation	No. of Time Periods	Reason for Inclusion
14/01/2013–18/01/2013	Days	5	Includes crash date (16/01/2013) and the two days either side.
16/01/2013–17/01/2013	Hours	48	To look at the hourly significance of the helicopter crash.

**Table 2:** Initial results from the space-time scan statistic model across differing temporal aggregations.

Time Aggregation	Total Number of Clusters Reported	Number of Significant Clusters (P<0.05)
Days	87	30
Hours	48	33

**Figure 3:**
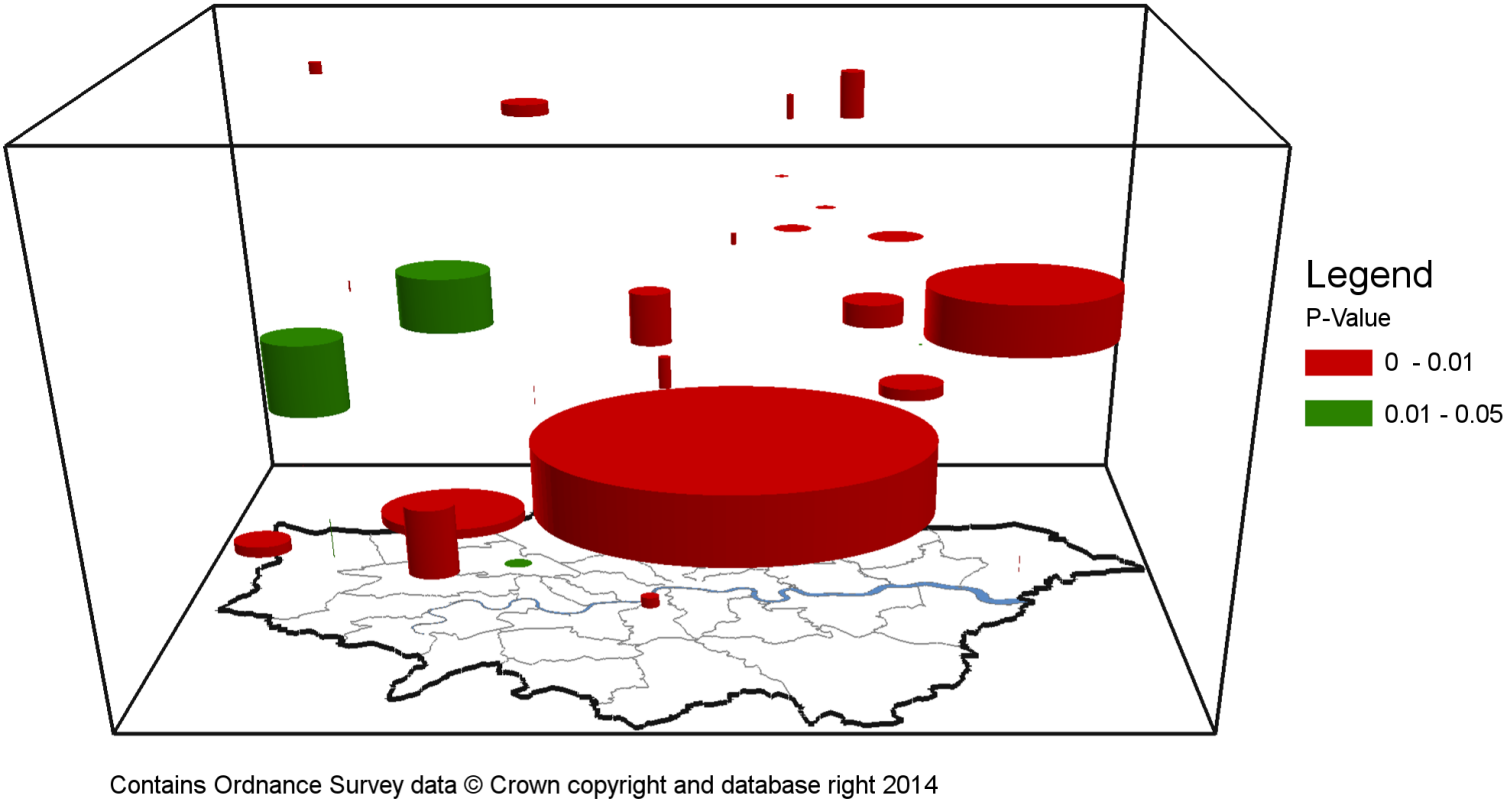
Significant hourly clusters within London between 16th January and 17th January 2013.

**Figure 4:**
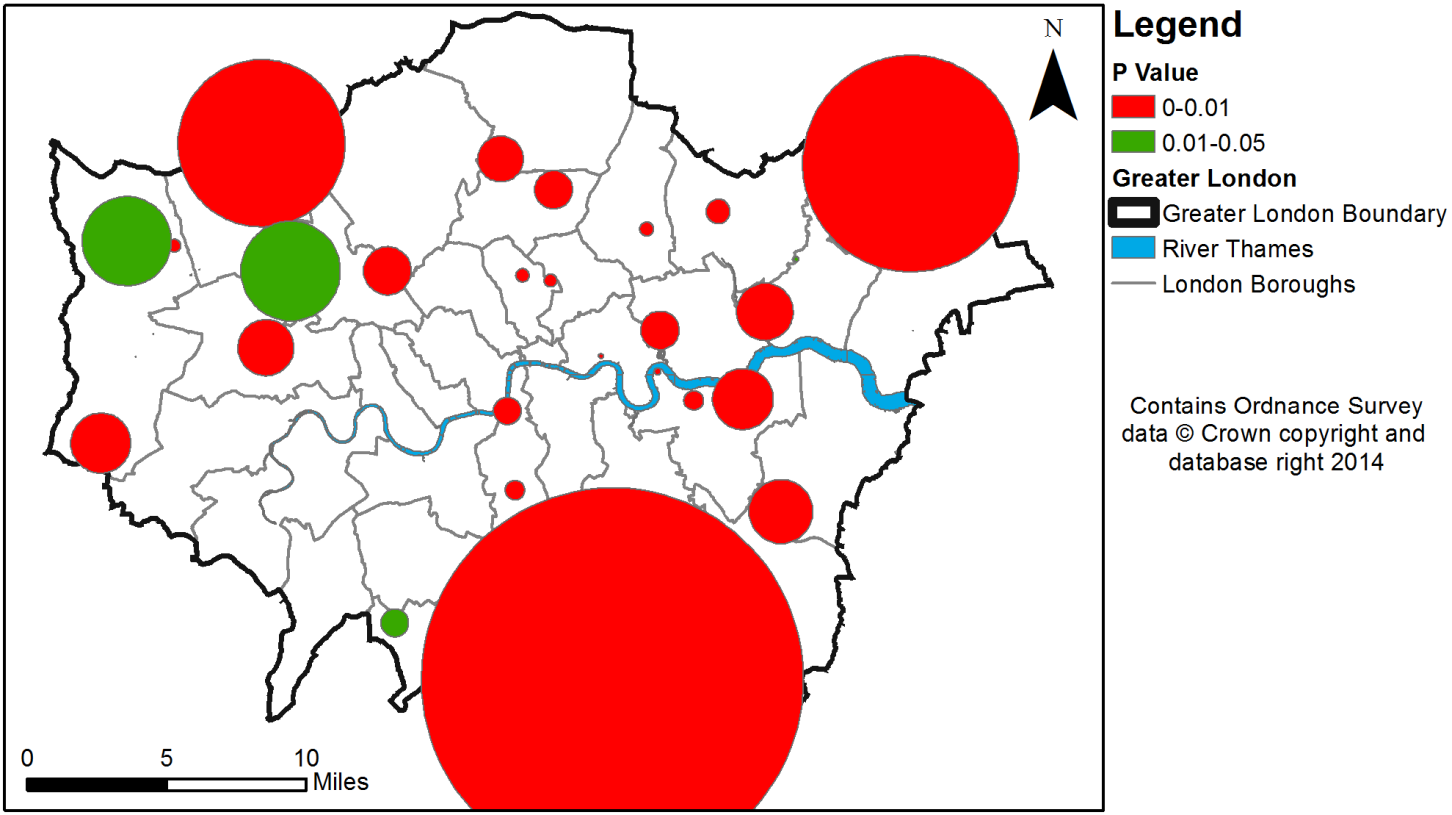
A top view of significant hourly clusters within London between 16th January and 17th January 2013.

Figure 3 maps the cluster outputs generated in an oblique view when using hourly aggregations within a space-time cube. Figure 4 is a top view of Figure 3, which shows the precise location and the size of these clusters in a 2-dimensional space. As it can be seen, clusters are spatio-temporally dispersed and display varying spatial and temporal ranges. However, these maps contain little useful information when trying to identify clusters related to the case study disaster event. In order to do this, clusters are classified into four descriptive topics using LDA. If at least half of the topics discovered are attributable to a space-time event, then it is assumed the tweet cluster relates to the real world event. If less than half of the topics can be attributed to an event, it is assumed the cluster does not represent a space-time event and is a spurious result. Table 3 provides the LDA topics generated for clusters which can be attributed to space-time events for daily aggregations, while table 4 provides the LDA topics for hourly aggregations. Terms deemed pertinent to the event are highlighted in orange while non-pertinent terms are highlighted in blue.

**Table 3:** Significant clusters identified during daily aggregations using Latent Dirichlet Allocation and their associated topics.

Cluster Event	Topic One	Topic Two	Topic Three	Topic Four
Arsenal V Swansea	Emirates	Swansea	Arsenal	Good
Chelsea V Southampton	Premiership	Stamford	Good	Bridge
London Helicopter Crash	Crane	Vauxhall	Helicopter	Thanks
New York Knicks Vs Detroit Pistonsat the 02 Arena	York	Knicks	Greenwich	Pistons
Train Delay A	Train	C2cRail	Away	Electricity
Train Delay B	Back	Hour	Thank	C2cRail

**Table 4:** Significant clusters identified during hourly aggregations using Latent Dirichlet Allocation and their associated topics.

Cluster Event	Topic One	Topic Two	Topic Three	Topic Four
Arsenal V Swansea	Arsenal	Swansea	Emirates	Stadium
London Heathrow Airport	Amazed	Back	Day	Airport
London Helicopter Crash	Vauxhall	Crash	Helicopter	Thanks
New York Knicks Vs Detroit Pistonsat the O2 Arena	Knicks	Arena	NYKnicks	Greenwich
Train Delay A	That	Announcement	Train	Electricity
Train Delay B	c2crail	Ham	Limehouse	F**k

As can be seen, both time aggregations pick up similar events. The exception to this is that only daily aggregations detect the Chelsea V Southampton football match, while only hourly aggregations detect a cluster relating to Heathrow airport. Crucially, both time aggregations detect a cluster relevant to the London helicopter crash, suggesting that STSS can be utilised to detect space-time disaster events using Twitter.

Looking at Table 3, three of the detected clusters represent sporting events, two represent train delays and one represents the helicopter crash. Some clusters, such as the NBA game, contain topics that are all relevant to the event; while others, such as train delay B, contain some words deemed relevant (“Hour” and “c2cRail”) and some noise (“Back” and “Thanks”). Accordingly, some tweets from this cluster may still be noise.

Studying Table 4, train delay B has two key terms based on location (“Ham”, based on West Ham, and “Limehouse”). Interestingly, the key term “f**k” emerges in topic four, suggesting some tweets within this cluster are less than happy with the train being delayed. In addition, it is noticeable that three clusters are found to have all four topics relevant to an event, suggesting these clusters contain very little noise compared to those found at daily aggregations.

**Figure 5:**
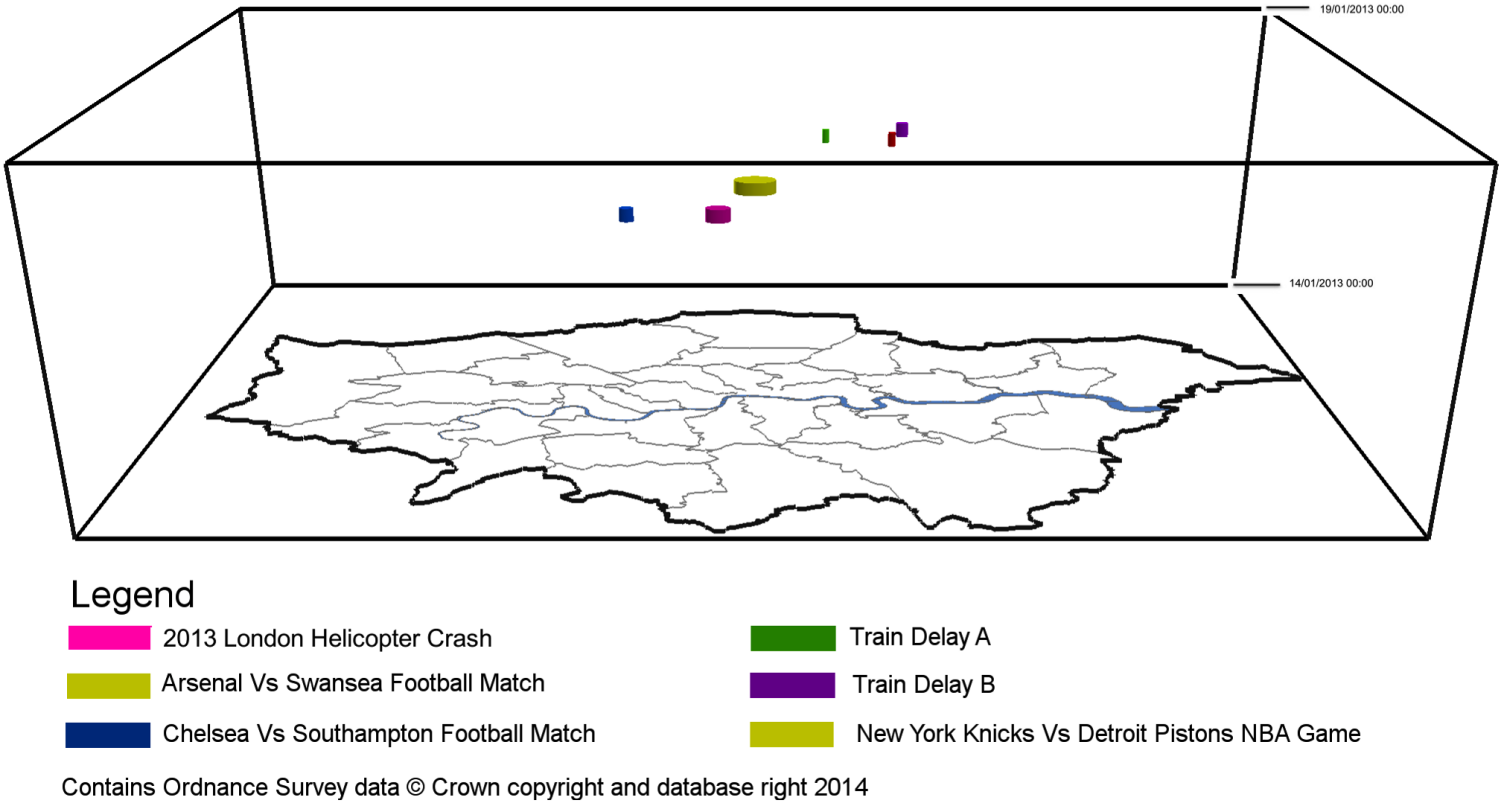
Daily cluster events identified within London between 14th January and 18th January 2013.

**Figure 6:**
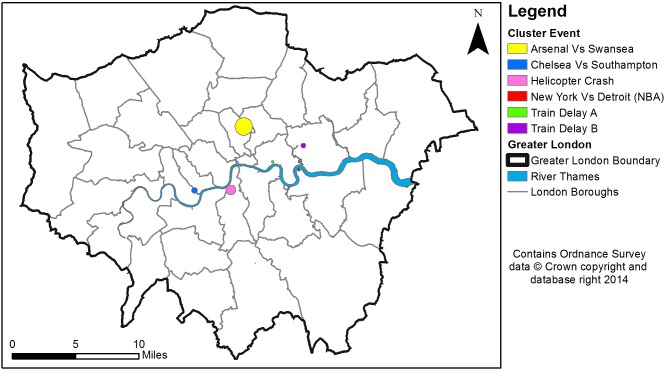
A top view of daily cluster events identified within London between 14th January and 18th January 2013.

**Figure 7:**
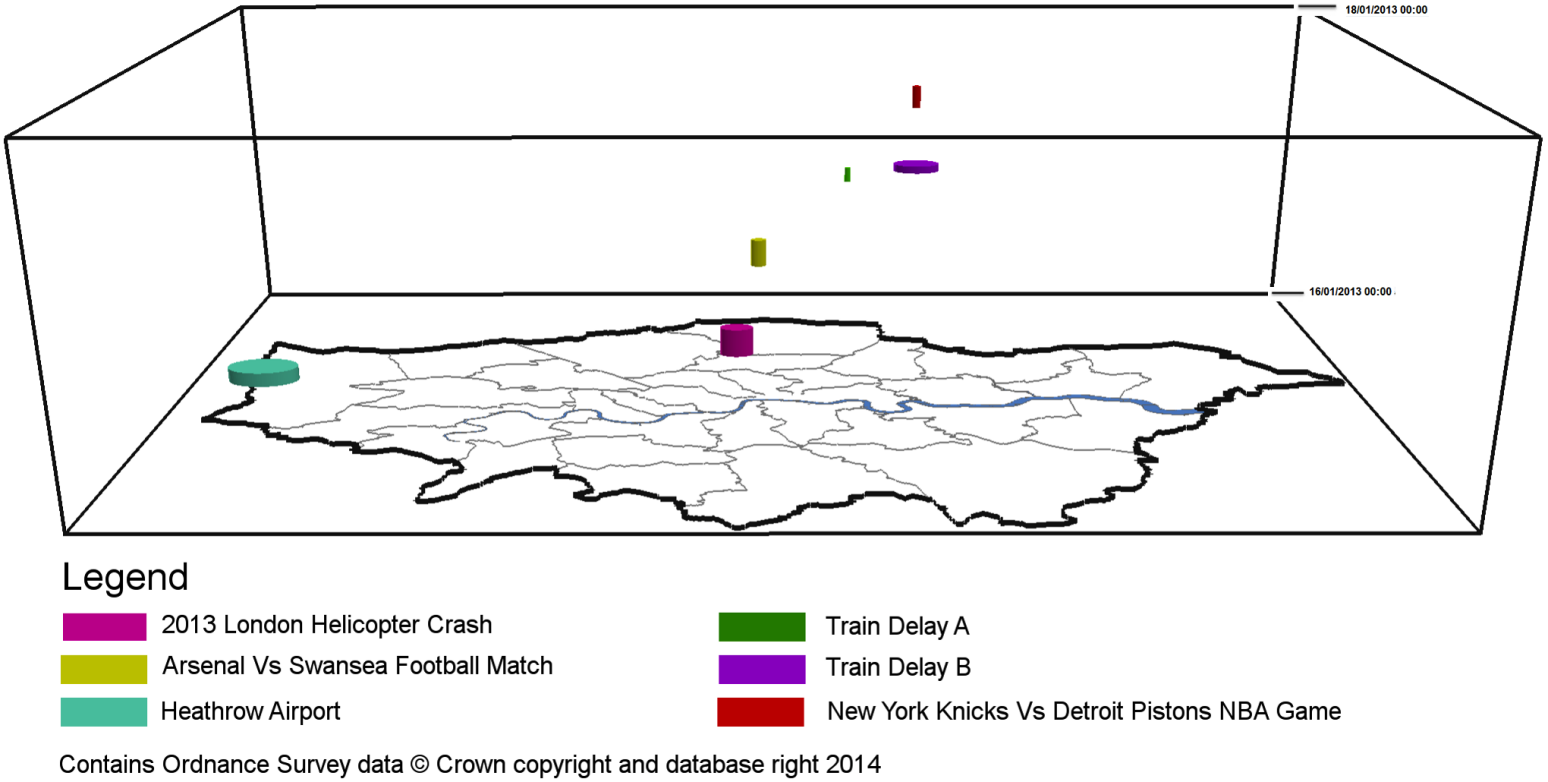
Hourly cluster events identified within London between 16th January and 17th January 2013.

**Figure 8:**
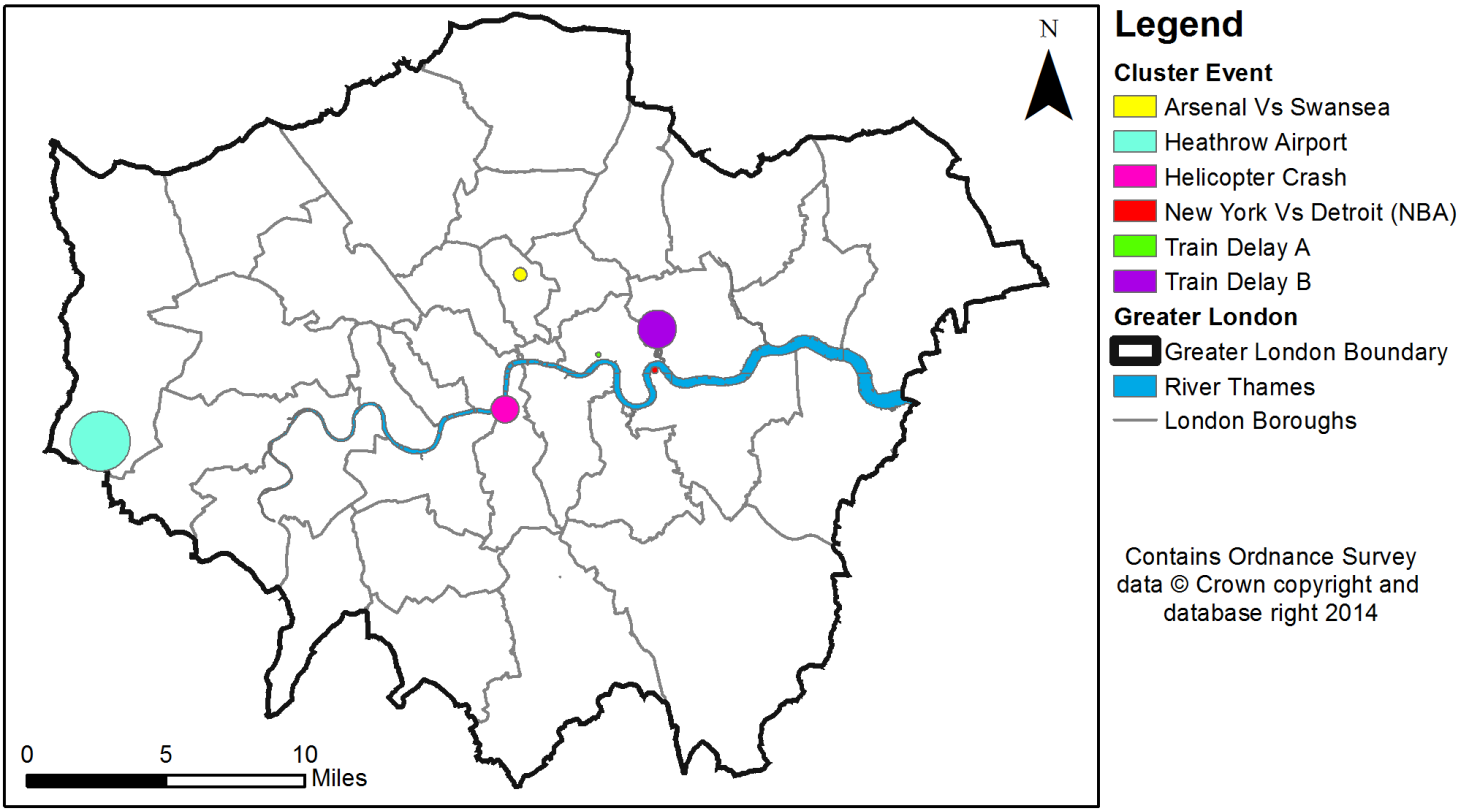
A top view of hourly cluster events identified within London between 16th January and 17th January 2013.

Table 5 provides the attributes of each detected significant space-time event, generated via STSS using daily aggregations, while Table 6 provides the attributes of those clusters detected during hourly aggregations. These clusters are then mapped in Figures 5–8, in oblique (Figure 5 and Figure 7) and top views (Figure 6 and Figure 8).

**Table 5:** Identified daily cluster attributes derived via space-time scan statistics.

Event	Latitude	Longitude	Radius (m)	Start Date	End Date	P-Value
London Helicopter Crash	51.4813	−0.1231	627.64	16/01/2013	16/01/2013	4.0×10^−15^
New York Knicks Vs DetroitPistons at the 02 Arena	51.5025	0.0024	187.19	17/01/2013	17/01/2013	7.7×10^−12^
Arsenal Vs Swansea	51.5534	−0.0965	1110.7	16/01/2013	16/01/2013	5.2×10^−9^
Train Delay A	51.5123	−0.0449	161.64	17/01/2013	17/01/2013	1.9×10^−4^
Chelsea Vs Southampton	51.4816	−0.1894	365.17	16/01/2013	16/01/2013	1.3×10^−3^
Train Delay B	51.5299	0.0119	300.0	17/01/2013	17/01/2013	1.2×10^−2^

**Table 6:** Identified hourly cluster attributes derived via space-time scan statistics.

Event	Latitude	Longitude	Radius (m)	Start Date	End Date	P-Value
London Helicopter Crash	51.485	−0.1239	806.19	16/01/2013 08∶00	16/01/2013 11∶00	1.0×10^−17^
Train Delay B	51.5247	0.0047	1109.6	17/01/2013 09∶00	17/01/2013 09∶00	2.3×10^−11^
Arsenal Vs Swansea	51.555	−0.1084	387.22	16/01/2013 17∶00	16/01/2013 20∶00	9.9×10^−9^
New York Knicks Vs Detroit Pistonsat the 02 Arena	51.5035	0.0018	195.25	17/01/2013 19∶00	17/01/2013 21∶00	1.1×10^−8^
Train Delay A	51.5123	−0.0449	142.84	17/01/2013 08∶00	17/01/2013 09∶00	1.1×10^−7^
London Heathrow Airport	51.473	−0.4622	1732.9	16/01/2013 05∶00	16/01/2013 06∶00	8.5×10^−4^

As can be seen in Table 5, no identified cluster spans more than one day. Importantly this would suggest that the London helicopter crash had little lingering effect within London or the Vauxhall area. In addition, it is interesting that no events are found on the 14^th^, 15^th^ and 18^th^ of January. Furthermore, Table 6 suggests that the cluster relevant to the helicopter crash only remained statistically significant for the four hours following the event. However, the cluster did become significant in the hour immediately following the crash.

## Discussion

The results found in this paper have confirmed that STSS can be applied to Twitter in order to detect significant space-time events. In this case, the event detected was the 2013 London helicopter crash disaster. This event was detected along with other non-disaster events, such as sporting matches and train delays.

However, perhaps the most interesting outcome of this research is the brevity of the significant cluster relating to the helicopter crash incident. When looking at the hourly duration of the helicopter crash cluster, it is seen that the cluster only remained significant for four hours. The cluster began in the hour following the crash (8 AM), indicating Twitter’s speed at reporting disaster events. However, by midday the cluster had lost significance, suggesting that the crash made little lasting impact on Twitter. This is surprising giving the magnitude of the crash. However, the lack of tweets may be explained by the fact that many local travel amenities such as bus shelters, train stations and roads were closed because of the crash [36], causing fewer tweets from the Vauxhall area. Somewhat unexpectedly, the cluster failed to span into the next day, even though the local area still saw heavy disruption on the following day [37]. One possible explanation is the presence of nationwide media reports creating tweets that were uniformly generated across space. This may have produced a purely temporal cluster of relevant tweets, which would not be picked up using the methodology put forward in this paper.

## Conclusions

This paper has aimed to identify a new methodology for identifying disaster events using Twitter data. This has been successfully achieved using STSS. It has been found that not only can STSS be applied to Twitter, but that abnormal space time clusters did exist within Twitter relating to the London helicopter crash disaster, as well as other space-time events such as sport fixtures and train delays.

This result means that future disaster events can now be detected without the issues associated with current event detection methodologies. Accordingly, future events can be more accurately detected, followed and responded to. However, further research is still required to improve upon this technique. Firstly, different disaster events should be analysed to ensure that STSS could be applied to varying types of disasters. Secondly, work should be undertaken exploring the possibility of applying prospective STSS to real-time surveillance of emerging space-time clusters. If prospective STSS is found to be applicable to identifying emerging events from Twitter, then the future of disaster detection and response could become more efficient, more dynamic and more powerful.
